# ASCO 2017—highlights of gynecological cancer

**DOI:** 10.1007/s12254-017-0371-z

**Published:** 2017-12-05

**Authors:** Bianca Radl, Brigitte Mlineritsch

**Affiliations:** 1 3rd Medical Department with Hematology, Medical Oncology, Hemostaseology, Infectious Diseases and Rheumatology, Oncologic Center, Paracelsus Medical University Salzburg, 5020 Salzburg, Austria; 2 Salzburg Cancer Research Institute with Laboratory of Immunological and Molecular Cancer Research and Center for Clinical Cancer and Immunology Trials, 5020 Salzburg, Austria; 3 Cancer Cluster Salzburg, 5020 Salzburg, Austria

**Keywords:** Ovarian cancer, Lymphadenectomy, Maintenance therapy, PARP inhibition, Advanced cervical cancer

## Abstract

At this year’s ASCO annual meeting several important studies in the field of gynecological cancer were presented. Here we report a personal selection of the most interesting and clinically relevant data.

At this year’s American Society of Clinical Oncology (ASCO) annual meeting several important studies in the field of gynecological cancer were presented. Here we report a personal selection of the most interesting and clinically relevant data.

Tremendous advances in the field of surgery in advanced and recurrent high grade serous ovarian cancers (HGSOC) have been achieved. The question whether a systematic pelvic and para-aortal lymphadenectomy (LNE) should be performed in addition to an optimal debulking surgery in patients with clinically node-negative advanced ovarian cancer was raised in the AGO LION trial (Philipp Harter et al. Abstract 5500; [1]). Neither median progression-free survival (PFS; 26 months in both arms; *p* = 0.30) nor median overall survival (OS; 65.5 months versus 69.2 months; *p* = 0.65) differed between the LNE arm and the non-LNE arm. As expected, more perioperative complications occurred in the LNE group. Based on these findings, a pelvic and para-aortal lymphadenectomy should not be routinely performed in clinically node-negative HGSOC.

The majority of patients with ovarian cancer will experience disease relapse. Palliative systemic treatment represents the standard of care at the time of recurrence. In the AGO DESKTOP III/ENGOT ov20 trial (Abstract 5501; [2]) Andreas du Bois et al. compared secondary cytoreductive surgery (CRS) followed by chemotherapy to palliative chemotherapy alone. If a complete resection was achieved, secondary CRS was associated with a significant impact on PFS (19.6 months versus 14.0 months; *p* < 0.001) in the preplanned interim analysis. Data concerning the primary endpoint (OS) are immature and will be reported with extended follow-up. Based on these results secondary CRS should at least be considered as a valuable option with a positive AGO score.

Results of the OvHIPEC trial, presented by W.J. van Driel in a poster session (Abstract 5519; [3]), demonstrated a benefit from the addition of hyperthermic intraperitoneal chemotherapy (HIPEC) to an interval cytoreductive surgery in patients. Patients received 3 cycles of neo-adjuvant chemotherapy, randomization took place intra-operatively, only those patients with a residual tumor smaller than 2.5 mm before surgery were eligible. Recurrence-free survival (RFS; 14.2 versus 10.7 months; *p* = 0.003) and OS (45.7 versus 33.9 months; *p* = 0.01) favored the additional HIPEC strategy. Due to the fact that only highly selected patients were included in this trial, the practical implication of these results are still under debate and therefore an additional HIPEC strategy remains an experimental approach and should only be performed within clinical trials.

In the field of systemic therapy of HGSOC encouraging results were achieved by maintenance therapy with the PARP inhibitors olaparib and niraparib as well as with cediranib, an oral antiangiogenic vascular endothelial growth factor receptor 1–3 inhibitor.

At the 2016 ASCO meeting, results of the SOLO-2 trial [4] demonstrated efficacy of olaparib in tablet formulation (with significant reduced pill burden compared to former capsule formulation as had been administered in the study 19 [5]). As maintenance therapy in patients with platinum-sensitive relapsed, germline, or somatic BRCA-mutated ovarian cancer, olaparib improved clinical outcome in terms of PFS (19.1 vs. 5.5 months, *p* < 0.0001), time to first subsequent therapy ([TFST];27.9 vs. 7.1 months), time to second progression ([PFS2]; not reached vs. 18.4 months) and time to second subsequent therapy ([TSST], not reached vs. 18.2 months).

J.A. Ledermann presented an update of the toxicity analysis (Abstract 5518; [6]) and Michael Friedlander of the health-related quality of life (HRQoL) data (Abstract 5507; [7]) of the SOLO-2 trial. The safety profile was consistent with the adverse event data that had been observed with the previously approved capsule formulation of olaparib of study 19. Most reported AEs were low grade (grade 1–2) and manageable. The majority of AEs (nausea, vomiting, and fatigue) were documented within the first three months of treatment initiation, whereas an improvement of symptoms was frequently observed during ongoing treatment. AEs leading to treatment discontinuation, such as hematotoxicitiy, were rarely observed (11%).

The SOLO-2 QoL analysis confirmed that olaparib maintenance did not negatively impact HRQoL relative to placebo and thereby, prolongation of PFS translated into significant symptom relief with benefit concerning HRQoL/time without symptoms of disease or toxicity [TWIST], 13.5 vs. 7.2 months, *p* < 0.001).

In contrast to olaparib, the benefit from maintenance therapy with the PARP inhibitor niraparib, as evaluated in the ENGOT-OV16/NOVA trial (Mansoor Raza Mirza et al. Abstract 5517; [8]), was independent of the BRCA mutation status in patients with platinum-sensitive recurrent ovarian cancer responding to platinum-based therapy. In the cohort with germline BRCA mutation (gBRCAm), PFS was 21.0 months in the niraparib arm versus 5.5 months in the placebo arm (*p* < 0.001). In the subgroup of patients with non-gBRCAm harboring a tumor with a homologous recombination deficiency PFS was 12.9 versus 3.8 months (*p* < 0.001), respectively, whereas in the overall non-gBRCAm cohort PFS was 9.3 versus 3.9 months (*p* < 0.001), respectively.

The three-arm phase 3 ICON 6 trial investigating cerdiranib in relapsed platinum-sensitive ovarian cancer was redesigned in 2011 due to low recruitment and was continued as a two-arm trial. Patients were randomized to chemotherapy alone or chemotherapy plus cediranib followed by cediranib maintenance therapy. The PFS (11.1 versus 8.7 months; *p* = 0.0001) and OS (27.3 versus 19.9 months, *p* = 0.21) results, as presented by J.A. Ledermann (Abstract 5506; [9]), provide evidence that cediranib extends PFS with a trend to an OS improvement, although the revised trial design was underpowered for OS analysis.

Alexandra Knipprath-Meszaros (Abstract 5515; [10]) evaluated the efficacy of an aromatase inhibitor maintenance therapy compared to observation after standard chemotherapy in combination with or without bevacizumab maintenance in order to delay recurrence in patients with newly diagnosed HGSOC with positive estrogen receptor expression. Twenty-four of 51 patients were randomized to aromatase inhibitor maintenance therapy with letrozole and 27 patients to the observation arm. The recurrence-free interval (RFS) was prolonged in the letrozole maintenance arm (1-year RFS 84% versus 65%; 2‑year PFS 74% versus 46%, *p* = 0.02). However, confirmation of these promising results in further trials is required.

In patients with stage III high grade endometrial cancer, a combination of radiotherapy and chemotherapy was superior to radiotherapy alone (5-year failure-free-survival: 69% versus 58%, *p* = 0.032; 5‑year OS 79% versus 70%, *p* = 0.114) in the PORTEC 3 trial as presented by Stephanie M. de Boer (Abstract 5502; [11]). No significant improvement of clinical outcome but significantly more toxicity with combined chemoradiotherapy was observed in women with earlier stage I and II cancers.

In the GOG 258 trial (Daniela Matei et al. Abstract 5505; [12]), women with stage III or IV endometrial cancer that had undergone an optimal debulking surgery where randomized to chemoradiation followed by chemotherapy or to chemotherapy alone. Postoperative chemoradiotherapy demonstrated a considerable effect on preventing nodal and local recurrence (5-year vaginal recurrence 3% versus 7%, 5‑year pelvic and para-aortal recurrences 10% versus 19%) without affecting RFS, probably due to a higher rate of distant recurrences (27% versus 21%) in the combination arm. According to these results, 6 cycles of adjuvant chemotherapy should remain the standard, but in selected patients the addition of radiotherapy should be considered with the aim to reduce the risk of local recurrences.

Last, but not least, promising data from the Checkmate 358 trial, a phase I/II trial, investigating nivolumab in patients with human papillomavirus-positive advanced cervical, vaginal or vulvar cancer, were presented by Antoine Hollebecque (Abstract 5504; [13]). Twenty-four patients were included in this trial; the majority of patients (*n* = 19) were diagnosed with advanced cervical cancer. Responses were only seen in the cervical cancer cohort with an overall response rate of 26% (5 of 19). After 6 months of follow-up, median duration of response was not reached in this cohort.
